# Creativity as action: findings from five creative domains

**DOI:** 10.3389/fpsyg.2013.00176

**Published:** 2013-04-16

**Authors:** Vlad Glaveanu, Todd Lubart, Nathalie Bonnardel, Marion Botella, Pierre-Marc de Biaisi, Myriam Desainte-Catherine, Asta Georgsdottir, Katell Guillou, Gyorgy Kurtag, Christophe Mouchiroud, Martin Storme, Alicja Wojtczuk, Franck Zenasni

**Affiliations:** ^1^Aalborg UniversityAalborg, Denmark; ^2^Laboratoire Adaptations Travail-Individu, Université Paris DescartesParis, France; ^3^Laboratoire PsyCle, Aix Marseille Université (AMU)Aix-en-Provence, France; ^4^Institut des Textes et Manuscrits Modernes, Centre National de la Recherche Scientifique ParisFrance; ^5^Laboratoire Bordelais de Recherche en Informatique, Université de BordeauxBordeaux, France

**Keywords:** creativity, action, art, design, science, scriptwriting, music

## Abstract

The present paper outlines an action theory of creativity and substantiates this approach by investigating creative expression in five different domains. We propose an action framework for the analysis of creative acts built on the assumption that creativity is a relational, inter-subjective phenomenon. This framework, drawing extensively from the work of Dewey (1934) on art as experience, is used to derive a coding frame for the analysis of interview material. The article reports findings from the analysis of 60 interviews with recognized French creators in five creative domains: art, design, science, scriptwriting, and music. Results point to complex models of action and inter-action specific for each domain and also to interesting patterns of similarity and differences between domains. These findings highlight the fact that creative action takes place not “inside” individual creators but “in between” actors and their environment. Implications for the field of educational psychology are discussed.

## INTRODUCTION

Creativity has been studied for more than a century and has recently been a subject of debate in educational psychology (Smith and Smith, 2010). This is because, despite the general consensus that we need more of it, especially in the educational system (Makel, 2009), creativity scholars are still struggling to understand the *nature* of this complex phenomenon and are quite far from designing highly effective programs for *enhancing* creative expression (for a review of education and creativity see Fasko, 2000–2001). Dominant models of creativity associate it with cognitive mechanisms (such as divergent thinking) and personality traits (like openness to experience) but fail, on the whole, to properly engage with the social and material aspects (with a few notable exceptions, e.g., Csikszentmihalyi, 1988). From an educational perspective, this omission is counterproductive as individualistic accounts of creativity place their emphasis on “inner” attributes that are either not fully developed in children or hard to educate (for harmful myths in this regard, see Plucker et al., 2004). Educational systems represent, in the end, a certain kind of environment and, if we are committed to understanding and stimulating children’s creative expression, we need a theory of creativity capable of articulating “internal” and “external” facets of creative expression at its different levels: from the most mundane (typical for the school environment) to the highly celebrated and visible.

Under these circumstances, the main purpose of the present article is twofold. At a theoretical level it advances a relatively novel conception of creativity in a landscape dominated by cognitive theories, that of *creativity as action and of creative work as activity*. Conceptualizing creativity with the means offered by the psychology of action, and in particular by pragmatist approaches to human action, leads to the development of a *situated model* of creative activity. Our second aim is to apply this model to interview data with recognized French creators from five different fields – art, design, science, scriptwriting, and music – leading to a complex image of human creativity both within and across domains of activity. As will be argued in the end, this theoretical approach has significant benefits for the field of educational psychology. A detailed analysis of creative action in the case of established creators can offer important insights regarding what facilitates or constrains creativity and, therefore, enable us to think about effective and domain-specific ways of stimulating creative expression in both children and adults.

### CREATIVITY IN AND AS ACTION

An inquiry into how creativity “takes place” is necessarily one that concerns itself with models of the creative process. Traditionally this process has been considered to be *mental/cognitive* in nature and *individual* in manifestation (see Glaveanu, 2010). Furthermore, the first models to be proposed excelled in depicting a rather orderly and simplified succession of stages for it, usually four. Wallas (1926) offers a classic example in this regard with his well-known distinction between preparation, incubation, illumination, and verification. Subsequent models added both complexity and dynamism to these initial proposals. Mumford et al. (1991), for instance, described a more elaborate succession of stages – problem construction, information encoding, category search, specification of best-fitting categories, combination and reorganization of best-fitting categories, idea evaluation, implementation, monitoring – and indicated many feedback loops among them. Botella et al. (2011) proposed a dynamic approach to the artistic creative process in which it is possible to “jump” some stages, to realize some simultaneously, and to go back to previous work phases. As Lubart et al. (2003) noted, early concerns with the creative process resulted primarily in stage models and generated sustained arguments about the exact number and characteristics of each stage. In contrast, more recent theories shifted the focus to *sub-processes *and the* micro-level dynamic* of creativity, conserving nevertheless a predominantly cognitive perspective on the phenomenon (e.g., Ward et al., 1999).

In contrast to purely cognitive models, action theories of creativity start from a different epistemological premise, that of *interaction and interdependence*. Human action comprises and articulates both an “internal” and “external” dynamic and, within its psychological expression, it integrates cognitive, emotional, volitional, and motivational aspects. Creativity, from this standpoint, is *in action* as part and parcel of every act we perform (see Joas, 1996). Creativity exists on the other hand also *as action* whenever the attribute of being creative actually comes to define the form of expression (and, as such, we can talk of “creative work” as different from other types of work which, in themselves, don’t completely lack the attribute of creativity). This particular understanding of creativity is not on the whole absent from past and present literature (see Glaveanu, 2012a). Woodman and Schoenfeldt (1990), for example, advocated some time ago for an interactionist model of creative behavior, one that starts from an understanding of the “organism-in-its-environment”. A strong link between creator and situation also characterizes Gruber’s evolving systems approach to creativity (see Gruber and Wallace, 1999) and its emphasis on ecological, longitudinal, contextual, and situated investigations. The creator is an evolving system within larger evolving systems (professional, social, political, etc.) and his or her action is always contingent on this dynamic co-evolution (see also Csikszentmihalyi, 1988).

But, in the end, what is action? The notions of act, action and activity have been theorized since the beginning of the past century by thinkers from a multitude of different schools of thought, spanning from American pragmatism to Russian cultural-historical psychology. Relatively dormant under the prominence of behaviorism and then cognitivism, they re-emerged in the past decades especially as part of social and socio-cultural psychology. In cultural psychology, the concept of activity is essential for understanding the development and manifestation of the human psychic in various cultural contexts (see Eckensberger, 1995; Cole, 1996; Ratner, 1996; Wertsch, 1998). Human action is defined by its intentionality and the mediation of various systems of tools, signs, and artifacts that make it comprehensible and symbolic. It takes place in a setting and involves both the organism, in its unity between body and mind, and a socioculturally constructed environment. Finally, action is often joint action and is both facilitated by and facilitates human social relations. These characteristics of action are present in John Dewey’s work, the leading pragmatist who, beside his writings on education, democracy, nature, and esthetics, was also an important theorist of activity (see Miettinen, 2006). From his rich intellectual legacy, we will be focusing here on one of his most celebrated works, “Art as experience,” first published in 1934, in order to reconstruct his vision of human action.

**FIGURE 1:**
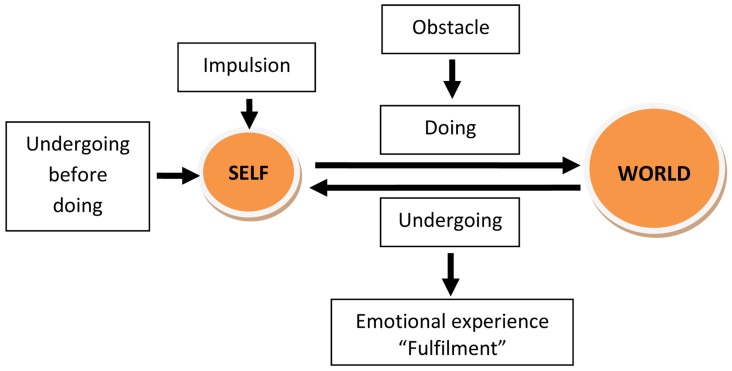
**A model of human experience (after** Dewey, 1934).

For Dewey, what brings action and creativity together is *human experience*, defined precisely by the interaction between person and environment and intrinsically related to human activity in and with the world. A graphic representation of his conception is offered in **Figure 1** below (see also Glaveanu, 2012b). Action starts, as depicted, with an impulsion and is directed toward fulfillment. In order for action to constitute experience though, obstacles or constraints are needed. Faced with these challenges, the person experiences emotion and gains awareness (of self, of the aim, and path of action). Most importantly, action is structured as a continuous cycle of “doing” (actions directed at the environment) and “undergoing” (taking in the reaction of the environment). Undergoing always precedes doing and, at the same time, is continued by it. It is through these interconnected processes that action can be taken forward and become a “full” experience.

The framework presented here is relevant for our understanding of creative action and Dewey himself has elaborated it in relation to art and the activity of the artist. The creator acts on the world in an attempt to materialize an artistic vision. However, this action is pared by a reaction from the world, one that the creator needs to undergo, to be aware of and integrate, in order to continue the work. In Dewey’s words (1934, p. 116), art:

“is a developing process. (…) the artist finds where he is going because of what he has previously done; that is, the original excitation and stir of some contact with the world undergo successive transformation. The state of the matter he has arrived at sets up demands to be fulfilled and it institutes a framework that limits further operations.”

As such, for Dewey, artistic work is not the outcome of the artist alone, and neither of the work of art. Creative expression is precisely “located” at the interaction between self and art object (Benson, 1993). Such a description resonates widely with many conceptualisations of artistic work (see Getzels and Csikszentmihalyi, 1976, discussion of the role of tension in art) and also with the experience of artists themselves. Israeli (1962) for example, in proceeding with a series of self-observations while painting, noted that “check and evaluation of the operations and outcomes are followed quite often by plans, suggestions, and decisions which control the subsequent operations on the painting” (p. 256). The continuous *cycle between doing and undergoing* that is at the core of Dewey’s conception seems to express a valid approach in the case of art, and, potentially, beyond it. It is argued here that the framework depicted in **Figure 1** has indeed a broader applicability in the psychology of creativity and constitutes, among other things:

• A model of the creative process based on cycles of doing and undergoing;

• An integration of behavioral, cognitive, emotional, and motivational elements;

• A re-evaluation of “impulsion” and “obstacle” as defining features of creative work;

• A contextual and relational account of human creativity.

### THE PRESENT STUDY

The study presented here focuses on creative activity within five creative domains: art, design, science, scriptwriting, and music composition. By applying the action framework proposed above, we explore the generalities and specificities of the doing-undergoing cycle in *each* domain and *across* domains. This framework can be potentially applied at three levels of analysis: a micro-level, focused on creative acts as they take place; a mezzo-level, concerned with the creation of a particular work or series of works; and a macro-level where the unit is the larger scale of creative activity, oftentimes the lifetime work of a creator. Considering the fact that we will rely on interview data, there is a possibility of uncovering elements belonging to all three levels but essentially, in lack of micro-level observations, the conclusions will be formulated at mezzo and macro levels. As such, the research is *exploratory* in nature and guided by the following general questions: What are the impulsions? What kind of obstacles do creators come across? What do they do? What do they undergo? How does the cycle of doing and undergoing actually take place? What are the main sources of “undergoing”: social, material, personal, etc.? Is there fulfillment and how does it contribute to the creation of full experiences? This investigation allows us to build more particular descriptive “models” for each of the five domains in terms of all the elements above and, by using the same scoring grid, to compare the creative action of artists, designers, scientists, scriptwriters, and composers.

## MATERIALS AND METHODS

### PARTICIPANTS

The sample consisted of 60 professional creators, 12 from each of the five domains, currently living and working in France. The main criterion for selection was for respondents to have extensive experience in their domain (overall, on average, the work experience ranged between 10 and 20 years). Demographic characteristics of the sample are presented in **Table 1**. As can be noticed, over two thirds of the participants are male, the distribution between sexes varying according to domain. Age also varies, the average for all domains except music being between 40 and 50 years.

**Table 1:** Demographic characteristics of the sample.

	Art	Design	Science	Scriptwriting	Music
Sex	7 m 5 f	8 m 4f	11 m 1f	6 m 6 f	10 m 2f
Mean age*	47	41	42	49	53
Age range*	35–66	24–60	28–57	40–63	36–63

* There were a few missing values for age: 3 for design, 4 for science, and 5 for m

All respondents had received higher education, in most cases directly specializing in their current profession. Important to note, the five groups are not completely homogenous in terms of creative output. Artists in the sample were primarily sculptors and/or painters, a few working with video and photography. Designers covered a more varied type of production, from decorative objects, interior design and furniture to visual communication, logos and packaging. The scientists group included six physicists (and astrophysicists), three mathematicians (theoretical and applied mathematics), two information and technology specialists and one chemist. Scriptwriters were more uniform, all writing film scripts for cinema or television. Finally, the musicians were composers working on either instrumental or electro-acoustic pieces.

### MATERIAL

The method used for data collection was represented by *semi-structured interviews* following a similar topic guide across domains. The interview started with a general presentation of the participant, continued with a description of his/her work and activity and, in the last part, invited a reflection on the creative process and the place of the creator and his/her domain in society. In particular, an adaptation of the critical incident technique (see Flanagan, 1954) was used in order to elicit more precise descriptions of respondent’s creative work. Interviews usually lasted between one and 2 h and were afterward transcribed verbatim for the purpose of analysis.

### PROCEDURE

The participants were approached and fully informed about the project before the interview took place. Their consent was registered and anonymity guaranteed. After the interview, all transcripts were subjected to thematic analysis (see Attride-Stirling, 2001) and the coding frame was *both* *theory and data driven*. The main analytical categories were offered by the framework presented in **Figure 1** (impulsion, obstacle, doing, undergoing, emotion) but their subcategories were defined after a preliminary coding of the first interviews from each group. As such, in the end, the coding frame included 11 codes, a summary of which can be found in **Table 2**. A similar coding frame had been elaborated and used successfully in previous research concerning creativity in craft activities (Glaveanu, 2012a,b).

**Table 2:** Coding frame.

**Code**	**Definition**	**Examples**
Impulsion	The motivation for action: why the person is doing a certain action	The need to create, to learn new things, to write, to express, to know (curiosity), to touch, etc.
Obstacle	Difficulties and/or limitations on the whole or at different stages	Lack of money, lack of time, lack of support, “inspiration block,” etc.
Doing – stages	The different stages or phases of creative work and how it advances	Documentation, first draft, maquette, prototype, final outcome, etc.
Doing – procedures	The different techniques creators use at different stages of their activity	Taking notes, using forms of brainstorming, using repetition, deformation, making associations, etc.
Doing – tools	The material tools used	Paper, pencil, brush, colors, wood, computer (different types of software), metal, glass, etc.
Doing – Time/place	When and where creative work is done	In the “atelier,” at home, at university, in the morning, evening, at all times, etc.
Undergoing – material	The relation to the physical/material environment	Constraints and properties of materials or the technology involved
Undergoing – social	The relation to the social environment and the nature of social interactions	With clients, colleagues, family, collaborators, critiques, audience; issues of social recognition
Undergoing before doing	Everything that prepared the creator for the work	Reading, discussing with others, preparing the instruments, studying, seeing exhibits, etc.
Undergoing final result	Perceiving and judging the final outcome	Looking at what came out, judging when and if it is finished, its quality, etc.
Emotion	Emotional experience at the beginning, during and at the end of activity	Sadness, happiness, excitement, satisfaction, depression, anxiety, joy, dissatisfaction, etc.

After finalizing the coding frame, a second coder, familiarized with the theoretical framework, applied them to all 12 interviews of the art group. On average there was 93% agreement between coders, with some discrepancies mostly for “undergoing – social” and “undergoing final result.” This led to refining the initial definitions and then to the application of the updated coding frame for the entire sample with the help of the qualitative analysis software Atlas.ti. Following this stage, all relevant quotations for each code were retrieved, separately for each domain, and summarized thematically by considering their content. For example, in the five domains, the “doing – stages” code included a series of different actions (such as documentation, sketches, creating the final product, etc.). Establishing the exact work phases in art compared to music composition, as well as their “order,” was done by reading all the material coded under “doing – stages” and retaining only convergent information (i.e., what most creators in the particular domain had in common). This allowed us to build general schemas of creative action for each domain, synthesizing findings from the main codes: impulsion, obstacle, doing, undergoing, (before doing, material, and social) and emotion. The schemas are presented and explained in more detail in the results section. Whenever direct or indirect quotations are used, they are indicated as such by mentioning the respondent code (A – artists, D – designers, S – scientists, L – scriptwriters and M – musicians; order numbers range from 1 to 12).

## RESULTS

### CREATIVE ACTION IN ART

**FIGURE 2:**
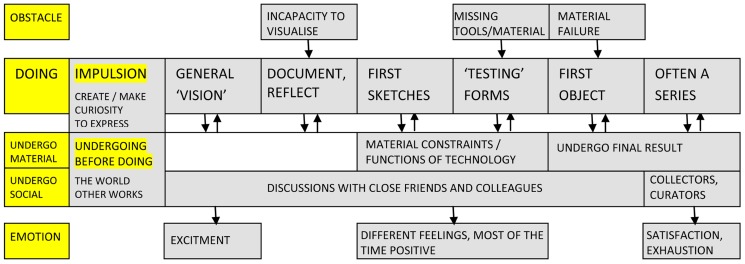
**Schematic representation of creative activity in the case of artists**.

As depicted in **Figure 2**, the creative activity of artists generally starts from the *impulsion* to create or make, to “do” or “incarnate” (A3), and it is also fed by a curiosity to see and understand (A6), to “find sensations” (A5), and a need to express (a “narrative desire,” A1). Artists refer often to their work as a “physical” necessity (A7) and to its motivation as a form of “internal pressure” (A9). This intense motivational drive meets certain *obstacles* when it becomes manifest, mainly the incapacity to visualize (A2) and reach a creative idea, missing the tools to work with (A5) and, toward the end, at times, the failure of material support (A10). Artistic activity seems to be defined, for most respondents, by a series of “crises” (A3), a constant self doubt and a desire to start everything afresh.

In terms of the *time and place* of their activity, most artists are very irregular, they don’t have specific working hours, mix activities and often get to work in different places. They take pride in not having “office work” (A5) although the most frequent place, at least for fabrication, is their studio. Several work at night, more as a matter of “germination” than urgency (A1). As for the general stages of their “*doing*,” the most frequent succession of stages is included in **Figure 1**. The whole process starts normally with a “vision” or idea. The “click” comes usually after a period of void, of wandering (A7), and the exact trigger can take many forms (for example an image, A9). This initial idea is nevertheless schematic, necessarily incomplete, and needs a time of reflection, documentation, incubation. These initial stages of “conception” lead to the stages of “fabrication” (A9), starting with the first sketches (e.g., the maquette) and up to a “draft” and the final object. Ideas are typically tested and experimented with on the basis of drawings and material depictions of the work, in clay or cardboard. The end result is often a series, as the first piece does not “exhaust” the sought after sensation (A6). Within these formal stages, there are numerous work *procedures* artists employ to realize their vision, including repetition (A8), multiplication (A10), permutation of elements and inversion (A2), simplification (A1), and change of medium (A9). Most of the time, the process is based on repetitive gestures, which for some are a means of relinquishing intentionality and “subjective decision” (A2, A4). Finally, the *material* medium involved is very diverse, including paper, pencil, computers, clay, metal, watercolors, acrylic, brushes, wood, plaster, latex, burin, ink, etc.

Before engaging with work, artists *undergo* family influences (A11, A12), and also formal, university training. The latter is considered by some not truly formative (A6), because most of the time inspiration comes from the world and the works of others. In the words of one respondent, “the first stage is life” (A1). The artists often consider themselves “sponges,” “90% of the time in a receptive state” (A4), allowing themselves to be “impregnated” by things and people (A11) who thus enter an inner “factory of fermentation” (A4). The walks, voyages, readings they make and exhibitions they see all nourish creative impulses. This is exacerbated by the “tactile” nature they seem to possess, where “observation goes through the hand” (A3). The *material undergoing* is marked by this “physical, sensorial, sensible presence” of the work, the “confrontation” with it (A6). Artists are always aware of and recognize material constraints, for example the chemical properties of pigments (A1). Objects “guide” the work (A10), they “speak” to the creator (A2), “call” each other (A8). In particular, objects resist the intentions of the artist. All of the sudden, they “ask a question” (A3) and very often “change the original plan,” being “stronger” than the creator, “imposing their rules” (A10). This is exactly what artists love about their work, this resistance, this reaction, this dialogue: the fact that the material all of the sudden says “wait, it is not just you!” (A12). Accidents enrich the project and one needs to constantly be on the alert for them (A5). But ideas also come from collaborators and the area of *social undergoing* is well represented. At times, the starting point of a project is an encounter (A4) and the entire process of work is collaborative. When this is the case, the partner is considered a “third eye” (A8) and the moments of discussion, even arguments, become a necessity (A1, A3). One needs “to see how others look at the work, to be able to see it as well” (A5). And “others” are also spectators and audiences. Social recognition gives pleasure and, whereas some confess showing “extreme permeability” (A4), others strongly affirm that their works is not meant to “seduce” (A7).

This multifaceted process of creation is unsurprisingly associated with different *emotional* states. The excitement of the creative idea is usually followed by a variety of emotions, while working. These range from pleasure and satisfaction to melancholia and even desperation but, most of the time, the reported states are positive and have to do with the “jubilation of being alive” (A6), the “pleasure of making” (A4) and above all the inner “certitude” (A9) when you are about to “do something” (A12), when the work starts “making itself” (A7). Confronted with the stereotype of the creator in turmoil, artists in this group were ready to contradict the myth and claim that they work to be happy and when happy (A10, A11, A12). The end of the creative process and *undergoing of the final result* are for all a delicate time when satisfaction mixes with exhaustion (A11) and the product is judged in terms of the initial vision and reaction of the audience. In a sense, some agree that an artistic object is “never finished” (A1), and take comfort in the perspective of having the work “back” and working it further (A9). This testifies to the continuity and cyclical nature of artistic activity, making the schema presented in **Figure 1** only a portion of a process filled with feedback loops, for working and reworking the work of art.

This *dynamic between doing and undergoing* is fully captured by artists who, in their interviews, often refer to their work as a series of “back and forth,” “come and go” (A2) between an initial imperfect and incomplete idea (A1) and external conditions that help the plan “mature” and keep it “flexible” (A4). Cycles of “action, reflection, action, non-action, plenty of action” (A7) describe the creation of art, during which the artist controls the process and at the same time lets it control the outcome (A8). This shapes the *experience* of art as something at the same time “rewarding and ungrateful” (A1) but, above all, defined as a “space of jubilation” (A2), of “extraordinary freedom” (A3) and of “pure magic” (A5).

### CREATIVE ACTION IN DESIGN

**FIGURE 3:**
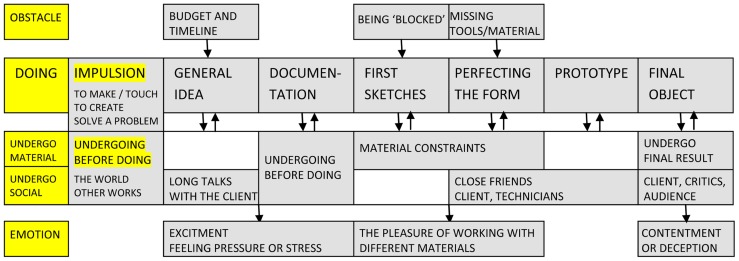
**Schematic representation of creative activity in the case of designers**.

Designers share some important similarities with artists, but also some clear differences, as can be observed from **Figure 3**. To start, the work of designers has its roots in similar *impulsions* to “make” things, to “build” (D3, D7), and also to “touch,” to work with one’s hands (D3, D8). The need to create and to be original and generate a “surprise” was also noted by some (D5, D3, D4), as well as the need to transform, change, and experiment (D2, D9). Specific to designers, they are also motivated by a desire to solve a practical problem (D3), and through this to respond to a certain need (D3), coming from a client. This presence, pressure and guidance offered by the client’s brief are almost universally mentioned. *Obstacles* reflect this to some extent and many discuss the financial and time constraints put on their activity (D3, D7, D11, D12), as well as “technical” difficulties (missing the proper technology; D6, D8, D11, D12) and being at times “blocked,” missing inspiration (D7, D8, D9, D10). Conflicts with clients are also mentioned as a source of stress (D7) as well as some self-imposed constraints (not to be too “literal,” D6; to strive toward simplicity, D7, etc.).

Similar to artists, most designers do not work every day according to a schedule and feel the need for some variation of their daily routine (D5). However, in their case, “availability” and external demands organize the work process and those who have a fixed studio work primarily from there (D3). Designers distinguish clearly between working for a client and working for oneself, in which the former is much more constrained and comes with some pre-set guidelines. The *stages of doing* can nevertheless be distinguished in both cases as starting from a general idea and gradually working toward its “concretisation” (D7). Just as in the case of artists, work starts from an idea (D4), an “intuition,” the “embryo” of the final form (D5). Certainly, whenever there is a client brief, the starting idea can be better defined, although this is not necessarily the case. The documentation stage is important, and, when time allows, quite extensive (D8 compares it to “going shopping” for elements). First externalizations of ideas take the form of drawings and sketches, and are progressively followed by 3D computer modeling (D4) and/or physical mock-up (D3, D9). On the basis of these steps, the form is perfected and several of its details are progressively defined (D10). The prototype stage can be optional (D2) but at times it is required, especially when there is scope for “industrialization” (D12). It is usually the case that designers present several ideas to the client (D7, D8, D12) and therefore several prototypes are made. The final object can require collaboration with technicians (D5) and an official presentation to the client (D11). The repertoire of work *procedures* involved in the stages above is extensive: amplification, deformation (D4, D12), association of ideas (D2, D8), use of allegories (D6), schematisation, and simplification (D7, D8, D11, D12), the re-use of shapes (D4), synthetic thinking (D9) and plenty of calculations (D11). Designers tend to have a notebook with them at all times, because ideas can come even in the middle of the night (D5). The range of *materials* used is also impressive, and includes, among others: paper, wood, cardboard, clay, metal, glass, textiles and cotton, steel, foam, leather, and plastic.

This attraction toward materiality was present for many since early childhood, when they engaged constantly in making or creating things (D7, D8, D9, D11, D12). As in the case of artists, the main sources of *undergoing before doing* are represented by the world and by other works. “Inspiration comes from everywhere” says D1, and the “starting point” is found while walking on the street, reading, taking the metro (D5). The important thing is to always “keep the antennas out” (D10), to be “attentive” and “open to the world” (D5), to “collect things” (D11) and “store them” in a “bank” of ideas (D9). “Creation is ultimately the reuse of a body of things that have been seen, read, digested, and it is the ability to re-fit, or to deliver, give life to this memory” (D2). Going to museums and exhibitions is a vital part of this process (D7, D10, D11) as one is “nurtured” by the work of others (D6). A special relation is set in place between the designer and the world of objects: “the designer is in the concrete” (D12). Forms of *material undergoing* are often mentioned in the interviews, from the need to explore materials, to “test their limits” (D3), to the frustrations one experiences when not “feeling” the fabric (D8). Material properties are to be discovered, to learn and re-learn with each new encounter. The maquette stage is particularly important for this, to “see what happens,” how materials “react” (D4). Only through these trials can the designer get to acquire “the intelligence of the materials,” to remember their solidity, rigidity, flexibility, or fragility, and to know exactly what needs to be used and when (D11). A designer’s creative activity is a game of constraints (D3, D4, D8), of “happy” accidents (D7), and moments of distancing and reflection (D9, D10). Distance is also achieved through the look of others, close friends, and collaborators (D3, D6, D9). However, when it comes to *social forms of undergoing*, it is the figure of the client that dominates. On the whole, designers seem to have an ambivalent relation with clients. Whereas some acknowledge the power of clients to decide how the work is done and when it is finished (D7, D8), others comment on the freedom of the designer, as constrained as it may be, to decide on the final form (D1) and propose alternatives (D4). Ultimately, there is a constant interaction with the client, back and forth exchanges (D1, D8), especially at the beginning and toward the end. Also, designers interact with technicians and engineers (D12) and with consumers (D6). In this context, some comment on the general lack of recognition for designers in society (D7).

Creative work is accompanied by different *emotions*. The beginning of work is exciting (D6) but can often generate stress and anxiety (D10) due to external pressure (D7). Usually the work itself is enjoyable (D6), marked by the pleasure of creating, of “making” (D9, D11). However, there can also be an anxiety for missed possibilities (D7) and a persistent doubt about the direction of the work (D10). The end brings satisfaction (D2), especially when the client is pleased (D10). An “artist” is never truly satisfied though (D1, D7) and the final product can generate “great surprise but also great deceptions” (D12). In general, the outcome is judged based on its esthetics (D11) and capacity to address the problem (D12). Its value is “relational” and so is its origin: “In the end, the project is a mixture of the original idea and then of the chances we came across, the meetings, so to speak, positive or negative, with materials, with techniques” (D5). The idea of the *dynamic between doing and undergoing* clearly emerges in this quote, and in all references to work as “trial and error” (D2), as going “little by little” (D3) in an almost “experimental process” (D4). The concept is there from the start but it is not complete, it changes (D1) and doesn’t yet have a form (D2). It is all finally “a permanent dialogue between myself and the object” (D10) that defines the very *experience* of design.

### CREATIVE ACTION IN SCIENCE

**FIGURE 4:**
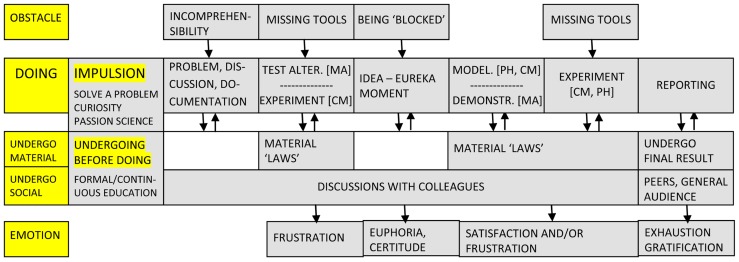
**Schematic representation of creative activity in the case of scientists**.

**Figure 4** depicts the activity schema in the case of scientists. There are notable differences from artists and designers. To begin, the *impulsion* that drives scientists toward their work mainly concerns the need to “solve,” to find an answer to a problem or question (S1, S2) and learn something new (S1, S4), coupled with great curiosity (S2, S5, S8). Many scientists mention also their passion for the domain of their choice and the pleasure they derive from working within it (S4, S6, S12, S7, S8), their need to go further in their domain (S7, S8), to arrive at new and different results (S5). A first *obstacle* for them is incomprehension or the inability to solve and understand (S2, S5), often associated with a feeling of being “blocked”. Missing proper tools is another major concern for scientists who depend on technology (S3, S5, S9, S10) and the mathematical apparatus (S7) to perform their research. All these difficulties can be traced back to the complexity of the phenomenon under study (S7) and for some, like astrophysicists, the impossibility of having direct access to certain physical realities, for example planets and stars.

The *time and place* for scientific activity tends not be fixed (S12) and some days are more productive than others (S1). However, unlike artists and designers, scientists are more committed to a stable working place, their university and their office, which is compared to a protective “cocoon” (S6). On the other hand, most refer to the continuous process of thinking as one can reflect on a problem from morning until late (S5). The actual *stages of doing* vary according to discipline but, overall, “there is the obsessive period, there is the enlightened period, and a period when you sweat hard to put things into shape” (S4). In more detail, and somewhat independent of the specific discipline, scientists start with a stage of discussion and documentation when the problem arises. Problems can come from anywhere, from articles, discussions with colleagues or attending seminars (S2) and their emergence is followed by substantial work in the library (S6). What is vital at this stage is for the “good questions” to be posed, because “in research, it is more important to find the questions than the answers” (S9). Then the work process becomes differentiated. In mathematics there is a long period of eliminating “false tracks” and, once the right idea “comes,” a demonstration for it needs to be set in place (S3, S4). In chemistry, experimental results prompt further questions and ideas, these are then modeled and again tested experimentally (S5). In theoretical physics and astrophysics, scientists collect observations, build a model and then test its assumptions (S1, S7). Finally, IT specialists in our group deal with creating computer systems and employ an experimental approach (S12). For all the scientists, however, a special stage is the idea or Eureka moment, usually “instantaneous” (S3), coming when least expected (S4). In contrast, the last phase of reporting, characteristic again for all, can be boring, tedious and non-creative (S1), “less amusing” (S4), although necessary (S7).

In terms of the *procedures* used, by and large scientists have two broad options in their work: to develop a new technique or use what already exists (S1). As for the second option, this can be done either by applying existing theories and models to recently discovered phenomena (S3, S5, S9) or by modifying or adapting procedures to fit the problem at hand (S3). More specifically, scientists strive toward parsimony (S7, S8), breaking down more complicated problems into simple ones (S10) and work by making connections between problems and domains (S6). Finally, when it comes to *materials*, the range seems more limited than that of artists and designers, being constituted primarily of books and articles (S2, S4), paper and pencil (S3, S4, S5). Computers and the Internet are “indispensible,” “omnipresent,” a real “right hand” (S1). To this we need to add experimental machines and technologies (S5) and lab equipment (S8).

As a precondition in science, all respondents experienced long years of formal education, of “apprenticeships” (S1) that helped them build their “general scientific culture” (S2). The *undergoing before doing* is completed by a more informal and continuous process of learning beyond one’s academic training. Reading books and articles, going to seminars and conferences (S2, S5, S6, S7) is fundamental. In all the fields above “you are always forced to learn something” (S4) and get to monitor and “devour” technical progress (S11). As previously mentioned, there is “enormous library work” before a study (S6) because you do not want to get to demonstrate what has already been demonstrated (S3). Discussions with colleagues supplement this effort and help ideas emerge or take shape (S7). At times, certain concerns and questions are “in the air” within the scientific community, and scientists pick them up and work on them (S1). This relates also to the notion of *social undergoing*.

Contrary to the popular image of the lone genius, if there is anything that defines scientific work it is the fact that it always happens with others, alongside others, in relation to others (S10). First points of contact are colleagues and peers, who play a crucial role in proposing problems (S4), formulating ideas (S6, S8, S9), clarifying them (S12), orienting the work (S2, S10) and finally evaluating it (S1). “There is a collective dimension and teamwork in the process of creation” (S5) and often it is the case that two or more people work together and have an ongoing critical exchange (S11, S12). In such circumstances, it becomes impossible in the end to know who had which idea (S7). Work “advances through meetings” (S4) and, at a broader level, scientific careers are shaped by the entourage and the chance of working with certain people (S5). The larger scientific community is a reality taken into account by all because scientific outcomes are there to be scrutinized and judged (S5, S8). There is a need for recognition (S6, S8) and, in an effort to gain status (S5), a scientist has sometimes to do a bit of “marketing” in promoting his/her work (S1). After all, there is “fierce competition” (S7, S8) in science, perhaps even more than in other creative domains. Another major source of undergoing comes from the *material world* and again the scientist’s activity is never as far from it as imagined. In physics, for example, there are laws, absolute laws, and “the phenomenon imposes incredible constraints,” “observable quantities” that defy all “creative calculations” (S1). Physics in this respect “guides the physicist,” and defines a precise space of possibilities (S7). Chemistry is not far from this because here as well a “game with matter” takes place (S5), and this matter resists and responds. As such, there is in science room for accidents and surprises, for unpredictable results (S9) and theories that need to adapt to the evidence of “experience” (S8).

And surprises also generate diverse *emotions*, depending on the stage and domain. Before getting the idea, a feeling of frustration often accompanies the search (S5, S8) and the Eureka moment is always associated with “excitation” (S4, S5, S7), an enormous satisfaction (S1, S10) close to euphoria (S3). This moment of certainty and inner clarity leads to a long period of testing and formulating that can be gratifying, when calculations go well (S1), but can also be associated with “suffering” during report writing (S4, S7). When work is finished, there is satisfaction and pride (S5) but also depression (S4) and anxiety about presenting it (S7). Overall, the ups and downs of scientific activity remind some of a manic-depressive state (S10), in which exaltation lives side by side with total exhaustion. This mirrors closely the general *dynamic of doing and undergoing* specific for scientific efforts, one in which “we advance, we are blocked, we reflect for a moment, we advance some more” (S2). A constant cycle of observation, modeling and testing (S7) takes place in science and shapes the *experience* of it, in which excitement and suffering are integral parts (S4).

### CREATIVE ACTION IN SCRIPTWRITING

**FIGURE 5:**
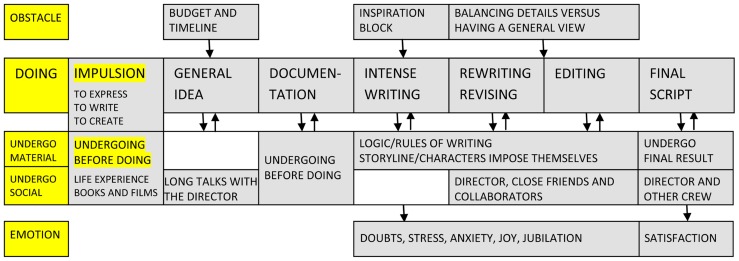
**Schematic representation of creative activity in the case of scriptwriters**.

The activity of scriptwriters, depicted in **Figure 5** resembles, to a certain extent, that of artists and designers. Fundamental for the *impulsion* of writers is their need to express (L2), to “tell” or “speak” (L5, L6), to show the world (L5) and “stage” something (L9). This is associated with a particular desire to write, a desire so strong that it makes some conclude: “if I wouldn’t write movies, I would write novels” (L6). Another important need is to create, to make something new and “provoke” others (L2). The act of writing or creating is never void of motivation because almost all respondents commented on the fact that they work with “ideas that evoke a desire” (L5), that “tell me something,” address the writer him/herself (L8) and this desire is to be clarified from the start. Finally, some have a more social motivational basis as well and feel the need to collaborate with certain producers or directors (L3, L10). This social basis is important because scriptwriting, even more than design, relies on a commission from the client. Consequently, one main *obstacle* is again represented by budget and timeline for completion. The world of cinema revolves around budgets for stories and “economic imperatives” (L3, L4, L8, L11) writers cannot possibly escape. Other difficulties have to do with inspiration blocks (L7, L8) and the complexity of gaining a comprehensive view of the entire work (L8, L10).

Unlike art and closer to science, scriptwriting requires a certain discipline and the *time and place* for work tends to be well established: writing almost daily (L4, L6), for at least 3 h (L1, L6), in an office (L7). This leaves space also for particular routines some have, for example that of writing in cafes (L1) or changing the program very quickly to foster spontaneity (L12). The *stages of doing* are also generally preset and they are learned as part of the craft. The process begins with a general idea, usually offered by the client. For some this idea needs to be quite structured (L5, L6), others enjoy more freedom at the start (L1) but in any case ideas are always worked closely with the film director (L11). After the topic of the scenario becomes clear, a stage of documentation is necessary, during which writers interview people and read books (L5), watch documentaries relevant to the subject (L12) and at times get to do some “fieldwork” (L10). Again, depending on the style of work, documentation can end up with a general plan or simply with material for further elaboration. A central phase of intense writing starts from documentation and leads into the editing stage. Writing “enormously” (L6) and using plenty of notebooks to put down ideas, sequences, even dialogs (L1) is crucial during this period. The outcome is usually very long and needs to be simplified and organized (L12), something that invites plenty of rewriting and revisiting. In the end, the plan (or sequence of episodes) is a “transitional object” (L10), perfectible and open to change. Finally, the editing part can take a long time in itself and it is when the dialogue and details for each scene are clarified (L4). Plenty of *work procedures* facilitate the task of writing a script. Among the “tricks of the trade” some mention writing a personal diary for the main characters (L1), or simply a narrative in first person (L2). Always having a notebook with you and taking a lot of notes is a requirement as well as taking regular breaks from the script to gain some detachment (L3). *Working materials* are relatively few in this case, primarily notebooks and the computer (L1). Interestingly, some love to write by hand (L7) and use the computer just for later transcribing or corrections (L10), whereas others put down on paper only the general plan (L9, L11).

The *undergoing before doing* for scriptwriters is largely based on personal life experiences and the enjoyment of books and films (L3, L5, L6, L7). Scriptwriters have habitually the attitude of a “hunter in the forest,” always on the alert, always taking notes (L10). Frequently, the starting point is connected to one’s own history, the things seen as a child, the lived experience and the people one knows (L6). At the same time, writers are “permanently nourished by the spectacle of the others” (L8). In the end, a scenario is always the “fruit of collaboration” and “it is never a solitary work, even if sometimes you work alone” (L11). Forms of *social undergoing* start with the client: the film director and producers. Writers need to comply with their wishes (L2), understand their vision (L5) and this requires constant interactions for establishing and maintaining a “common ground” (L1, L8, L9). “The relationship with the director is at once something intimate and devouring” (L10). In the end, the director’s views matter and he or she is the one to say when the script is “ready to go” (L1, L4). Close collaboration can also exist with fellow scriptwriters as, quite often, a script is a “shared work” (L5). A “ping-pong game” (L2) of “back and forth” (L10) starts between collaborators and their input is valuable because it can give perspective, “prevents one from turning around in one’s own madness” (L3). Friends are sometimes also used for this purpose (L1, L5, L7). By the end, everything is “co-written” between fellow writers and there is no way of knowing anymore who wrote what (L8). The relation with critics and the public can bring joy or suffering but rarely affects the work directly (L1, L6). Recognition is desired (L1) but the film industry is often plagued by jealousy and competition (L6) and too much praise or too much criticism can be equally blocking (L7). On the whole, there is rarely a real appreciation in society for the role and contribution of the scriptwriter and this is experienced by many as “humiliating” (L10).

Although not working with physical objects like artists, designers, and scientists, writers are by no means free of *material forms of undergoing*. On the contrary, these are equally present and directive in shaping the work flow. There is a materiality of the script and a moment in the process where it seems to take “a life of its own” (L2), when “the logic of the story is gradually unfolding” (L3). This moment is essential and needs to be captured because it signals that the project is on the right track (L1) and is taking the lead (L11). The characters have an important part to play in this unfolding given that, as they develop, they gain in power, become “alive” (L6) and start following a logic that imposes itself (L7). In a conflict between structure and characters, it is the characters that usually win (L12). This is part of the “laws” of dramaturgy – norms that generally guide the construction of the story (L5). Ultimately, another constant form of material undergoing has to do with re-reading the script, normally out loud (L8, L10), and sometimes by acting or miming the scene (L10).

The *emotional background* of scriptwriting is extremely mixed. Whereas making the plan can be both exciting (L9) and frustrating (L8), during the writing episodes a combination of pain (L5), anxiety (L11, L12), depression (L8), anguish (L4) and at times “intense joy” (L1), happiness and jubilation (L2), takes center stage. However, as repeatedly acknowledged, “when you love cinema there is always a pleasure to some extent” (L11). This feeling is exacerbated toward the end when relief and accomplishment are equally felt (L9); when everything is done and everyone is happy: “for 48 h I am the happiest woman on Earth!” (L1). Indeed, this emotional state is also an important criterion to evaluate the final outcome – the script is finished when satisfaction outweighs frustration (L7). This happens though after a continuous *cycle of doing and undergoing* when “you write something, you have it read, you re-write, you have it read” (L5), a back and forth movement (L11) of a specific nature and yet described by creators from all domains. The *experience* of scriptwriting makes no exception and confronts the creator everyday with his or her limits (L1), but, just as in every other case, “without contradiction there is no fulfilment” (L5).

### CREATIVE ACTION IN MUSIC COMPOSITION

**FIGURE 6:**
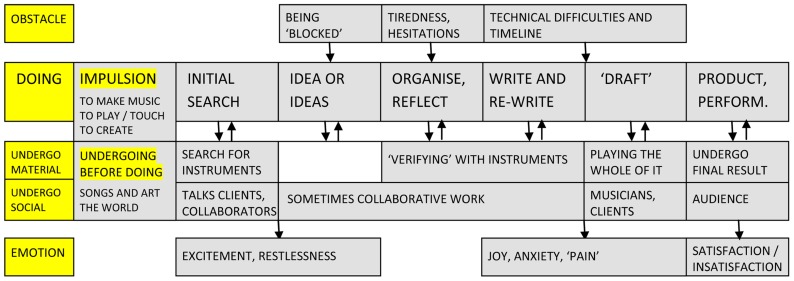
**Schematic representation of creative activity in the case of composers**.

In many regards, the group of composers shares similarities with other “artistic” domains (see **Figure 6**). To start, their *impulsion* is defined by a need to create music (M8), to work on a song (M2) or a particular musical element (M6, M8). As is the case with other artists, this work is seen as a “necessity” (M6), an inner “creative force” that imposes itself on the composer (M11). There is also a more precise need to “touch” and play an instrument (M2) and to be original, not necessarily in doing something never seen before (M6, M7), but something unknown to the author (M8, M9, M11). The nature of the *obstacles* is also shared with other domains. First comes the situation of being “blocked” (M1), the anxiety of the “blank page” (M7), when things stop being “fluid” and become difficult (M6, M10, M11). Then come tiredness (M1), hesitations, and constant questioning of the work (M6). Finally, in accordance with designers and scriptwriters, there are also timelines to be considered because most pieces of music are in fact commissioned by clients.

Similar to other artists, musicians as well discuss the irregularity of creation in terms of *time and place*, the fact that you cannot have a strict schedule (M1, M4, M8, M9) and depend on a specific state of “disponibility” (M6). Whereas some like to work in different places (M6), others have a studio (M10, M11). A particularity for this group has to do with the number of people who claim to be working at night (M4, M6, M9, M10, M11). The *stages of doing* usually start with an initial search. This is the case because very often for composers, even when working for a client, the preliminary guidelines are quite general (M7, M8). For this reason, composers are left to establish their own “sound palette,” looking for “sound actors” that will later be placed in a corresponding “script” (M4). Writing them down, the composer is then waiting for an “idea” (M8), for a “click” (M4). This is the second stage, when insights emerge out of an “accumulation of things” and one idea attracts another (M1, M3). More often than not, musicians work with ideas in plural, rather than one single “vision” (M8), as compositions have a time dimension. Given that there are many ideas or themes to work with, a moment of organization and reflection becomes necessary: distancing yourself a bit (M1) and trying to make a plan (M7). The writing and re-writing of compositions is of course a central stage in the production process, a technical phase (M8) when things become more “precise” and new ideas can spur from the process of writing itself (M6). Just as in the case of designers, composers often work on several versions of a song (M9). The “draft” stage is equally dynamic because editing takes place as the author listens to the work during rehearsals (M6, M11). Even in the final product phase, small changes can be made while the song is transcribed (M7) and the end truly comes when the music is officially recorded or played (M10). The whole process can be defined as a “progressive concretisation” (M7), from conceptualization to final performance (M6).

*Work procedures* guide activity and relate mainly to simplification (M1), varying instruments (M2) and themes (M3, M9), reposition and juxtaposition (M3), repetition (M3, M9), decomposition (M2), combination (M7, M9), etc. Many of these procedures are facilitated by the use of technology that permits the integration of effects, insertion, mixing, dividing, synthesizing, modulating and multiplying segments, compressing and decompressing, cutting and reorganizing (M5, M9, M11). It comes as no surprise that, among the *material tools* used by composers, computers, and mixing software are a priority (M1, M3, M4, M9, M10). However, instruments are also mentioned, along with more classical equipment: microphones, speakers, tapes, and even the traditional paper and pencil for writing music (M7).

Before getting to use these specialized tools though, and in order to be able to write music, a period of formal training is needed, sometimes starting from teenage years with playing certain instruments (M4, M9). The *undergoing before doing* is, in this case as well, continuous and “learning never ends” (M8), an integral part of it being listening to music of diverse genres and origins (M6, M7). Old songs offer inspiration (M1, M9) and excellent starting points for the initial search phase (M5). The world more generally is another great source of inspiration, and composers “love to look at things” (M5), to go to the cinema (M4), to read, paint and go to concerts (M6). They are often very curious, “receptive” (M7), like a “sponge” in absorbing their surrounding (M5) and adding things to the “reserve of what has been lived” (M8). Their relationship to the material world is particularly strong and reveals various forms of *material undergoing*.

To begin, musical notes and instruments have a materiality that is impossible to ignore. Notes are “compulsory” (M3) and instruments offer a framework of possibilities (M6). There is a very “primitive, tactile” feeling for all those who get to work with music instruments (M8), where the “immediacy” of the instrument (M9) needs to be mastered, practiced, “domesticated” even (M12). Another form of undergoing is embedded within the work process when composers write and then listen back, deciding what to do next with the material (M1, M2, M3, M9). This feedback is alimented by a “need for contact,” a need to “verify” (M7) one’s intuitions. Accidents play also a role in this process, and they are “artistically interesting to have” (M2, M12). In the end, music is “physical, it vibrates in the body” (M3) of the composer in ways that gradually become internalized, constituting an inner “voice”. On the other hand, music is also a social enterprise, and *social types of undergoing *link composers to clients, colleagues, interpreters/instrumentalists, and the larger audience. Clients impose particular constraints (M1) and can be hard to please so one has to work “in reaction” to propositions, trying to always bring a personal note (M7). With interpreters/instrumentalists the relation can also be fruitful but difficult. Compromises need to be made and, at times, complicated pieces are simplified (M6). With other colleagues there is a constant exchange of ideas and reflections (M4, M5, M12). For some, being a musician means working “in a group” (M9), where both success and failure are shared (M6). This helps to perfect one’s own technique because “composition as such is not taught, it is learned through encounters” (M11). Finally, the public is the final recipient of the work so the appreciation of others is part of the success of the composition (M4, M9) and critical feedback needs to be taken into account (M1, M11).

The final satisfaction or dissatisfaction with the outcome is relative to the public but also to the composer’s own evaluation of the result according to compositional criteria (M6) such as “coherence,” “good form” and “continuity” (M9). However, many respondents admit to never being truly satisfied (M7, M8) whereas others allow themselves to feel exaltation (M3) or happiness (M11). Other *emotions* characteristic for this work are the initial excitation and incertitude (M1), followed by different emotional states while working: plenitude (M1), pleasure (M2), “erotic sensations” (M3), anxiety (M7), “romantic sadness” (M8), jubilation or melancholy (M10), etc. These reactions come as a consequence of the particular ways in which *doings and undergoing interact*, their specific moments of “back and forth” (M10, M12), alternation between “zoom and distance” (M6), between gesture and listening (M5). Everything in music composition seems to be under the logic of “groping around” (M8, M9), of making and re-making that lead to a “spiral” of progress from one stage to the next (M6). What is interesting for the composer is “what is born out of the interaction with the tool, with the instrument, with the context” (M12). The *experience* of music creation is constituted directly by interaction and resistance which are necessary to “measure the value of one’s inspiration” (M10).

## DISCUSSION

The present article aimed to make a contribution toward developing an action analysis of creative activity. Grounded in pragmatist accounts of action, the framework proposed here focuses on the permanent exchange between a creator’s “doing” and the reaction it generates from the social and material world, the awareness of which is defined as “undergoing.” This broad perspective became gradually specified and resulted in the elaboration of schematic representations of creative activity in all five domains under investigation. Important to note, these schemas reflect *content-specific aspects* of activity for each field and not general and abstract creative processes. As such, depictions both confirm and expand previous results from the literature.

In *art*, for example, Mace and Ward (2002) proposed a basic succession of interconnected stages in the form of artwork conception, idea development, making the artwork and finishing the artwork/resolution. Similar moments in the artistic process are depicted in **Figure 2**: the general vision can be related to artwork conception, the documentation and reflection stage to idea development, making the artwork corresponds to the first sketches and their testing whereas finishing the artwork leads to the final moments of the “draft,” final product or series. Both conceptions seem to intersect in the claim that “the genesis of an artwork arises from a complex context of art making, thinking, and ongoing experience” (Mace and Ward, 2002, p. 182). In design, some current models of the creative process – such as the A-CM (Bonnardel, 2000) or the F-B-S model (Gero, 1998) – try as well to integrate components related to situated cognition. In addition, Tan and Melles (2010) have recently approached design work through the lenses of activity theory. Their description of observed activities as “for the most part dynamic, iterative, and opportunistic” (Tan and Melles, 2010, p. 474) corroborates previous descriptions of design activities as opportunistic (see, for a review, Visser, 1994) and matches the type of processes comprised in stages like “first sketches” and “perfecting the form” (see **Figure 3)**.

Investigations of *scientific* creativity for the most part either confirmed the classic four stages model of preparation, incubation, illumination and verification (see Sriraman, 2004), or enlarged it (see Busse and Mansfield, 1980). The model we propose here departs in a significant way from this traditional conception. Whereas the idea/illumination moment seems to be consistently mentioned by most scientists, the process is focused more on incremental progress from experimentation to mathematical formalism and then again experimentation. The same kind of gradual development was proposed by Csikszentmihalyi (1996) in relation to creative *writing*. Rather than one great moment of illumination, the author suggested a more continuous activity of generating “smaller” ideas, then connecting and revising them. The stages of “intense writing,” “rewriting/revisiting,” and “editing” reflect this insight. At last, descriptions of *music composition* by Bennett (1976) are similar to the ones proposed in **Figure 6**, starting from a “germinal idea,” continued with brief sketches, a first draft, elaboration and refinement and then completion. What action analysis brings to this field, though, is a greater acknowledgment of the role of social and material factors for composition. Kaschub (1997) once stated that restrictions and limitations play a key role in music creation. The origin of these restrictions and their result are two fundamental concerns for activity theory.

One of our declared aims in selecting five creative domains and using the same action coding frame for all the groups was to uncover possible patterns of similarity and difference between them (in agreement with current understandings of creativity that consider both its domain-general and domain-specific aspects; Lubart and Guignard, 2004; Baer and Kaufman, 2005). Such patterns are briefly presented in **Table 3** in terms of the main codes of impulsion, obstacle, doing, undergoing (material and social), and emotion. What can be immediately noticed is that, against a common presupposition that science would stand out and that design would “mediate” between it and the other three more “artistic” domains of art, scriptwriting, and music, we are confronted with a *patchwork of similarities and differences* between domains regarding each of the six criteria.

**Table 3:** Summary of patterns in creative activity in the five domains.

	**Art**	**Design**	**Science**	**Scriptwriting**	**Music**
Impulsion	***Create/express***	***Create****/solve*	*Solve/curiousity*	***Create/express***	***Create/express***
Obstacle	***Tools/material***	*Budget/****tools***	***Tools/material***	*Budget/time*	***Tools****/time*
Doing	***Idea/work/idea***	***Idea/work/idea***	*Work/idea/work*	***Idea/work/idea***	***Idea/work/idea***
Undergo (MAT)	***Physical prop.***	***Physical prop.***	*Laws/norms*	*Laws/norms*	***Physical prop.***
Undergo (SOC)	***Colleagues***	*Client*	***Colleagues***	*Client/****colleagues***	*Client/****colleagues***
Emotion	***(DIS)Satisfaction***	***(DIS)Satisfaction***	***(DIS)Satisfaction***	***(DIS)Satisfaction***	***(DIS)Satisfaction***

For impulsion, indeed, the three “arts” can be grouped under a general need to create and express, which somehow differs from a scientist’s urge to solve and learn about the world, designers sharing here a bit of both. Obstacles though bring art and science together in facing difficulties related to materials and tools; the problem of adequate tools is present also for designers and musicians. Budget and time are more pressing issues for scriptwriters, and resonate as well with the budget constraints of designers and the deadlines faced by composers. Across all domains, the “inspiration block” can be a common obstacle. The “doing” element reorders the five domains, this time along the lines of a dichotomy between scientists and other creators. If in science a dynamic seems to be set in place in which a general problem is examined, this work leads to an idea and the idea is developed in subsequent work. Artists, designers, scriptwriters, and composers all mentioned the idea, “vision” or client’s brief as the starting point. This initial input is processed and then further ideas emerge. Material forms of undergoing revolve mainly around the physical properties of objects for those domains which are immersed in the material world (art, design, and music), and around the laws and norms of the physical or dramaturgical universe for science and scriptwriting, respectively. From a social perspective all creators, independent of their particular discipline, emphasized the necessity of relating with others, exchanging ideas and being evaluated. The figure of the client is paramount in design and important for scriptwriters and composers as well, whereas colleagues or peers are regular interlocutors in science and art. In the end, the emotion dimension did not yield any significant differences between creators and it seems that, irrespective of domain, creative activity is marked by ups and downs, by oscillations between euphoria and depression, between satisfaction and dissatisfaction with one’s work.

Such findings are important for the educational field. To begin, they point to the fact that educating children for creativity should consider the domain specific features of creative action. It is certainly the case that, at school, children are not in the position of acknowledged artists or composers during their art and music classes, nor are they scientists who could make significant contribution to a domain while studying math or physics (or at least the probability is very low). It is widely accepted today that acts of historical or Big C creativity require many years of training, something formalized by Hayes (1989) as the “ten-year rule.” A crucial question, however, is what exactly happens during these years of training, many of which take place as formal schooling in an educational setting (see the example of scientists in our study). Also, how can this period of preparation – whose length again varies depending on domain – be most fruitfully organized to facilitate high-level creative expression? Moreover, because practice or preparation work are actually continuous for creators in most fields of activity (and certainly in the five domains studied here), we need to consider creative action as equally continuous and not taking place only when (and if) a highly celebrated outcome is actually produced. Under these circumstances, educators should focus on the nature and quality of what we called here “undergoing before doing” – the stage of preparing oneself for creative activity on the long run but also before working on particular projects. Ideas about what motivates recognized creators to work (found under “impulsion”) can suggest the kinds of needs and impulses we should encourage in children, from an early age. Finally, knowing about the stages of doing in particular domains can help us structure our teaching of artistic and scientific disciplines and make good use of those material and social conditions that facilitate creative expression (adequate tools, social recognition for one’s work, etc.).

In the end, it is also important to realize the shortcoming of the present research. To start, we reported findings here from a relatively small number of participants (although adequate for a qualitative investigation) and all belonging to a particular cultural context. We can also question to some extent the trustworthiness of self-report data, even though self-report scales are quite popular in creativity research and Hocevar(1976, p. 455), in another context, claimed they are “perhaps the most easily defensible way to identify creative talent.” But the most notable limitation, from a theoretical perspective, relates to our effort of translating theoretical assumptions into research devices. Going back to the psychology and philosophy of John Dewey, what transpires from all his writings is an effort to transcend dichotomies, especially those between self and world, and artificial segmentations between cognition, affect, motivation, and volition, all understood as building blocks of human experience. For analytical purposes though, segmentations had to be made, even temporarily, in order to end up with a broader, more dynamic and unitary picture of creative activity. While a certain dynamism was introduced by relating the doing of the creators and the obstacles they face to material and social forms of undergoing, on the whole, the schemas presented above do not contain many feedback loops *between stages*, as other models rightfully do (e.g., Mace and Ward, 2002). This limitation can be accounted for by the nature of interview data and the fact that interviews alone only offer verbal reconstructions of creative work and are thus subject to narrative formats (progressing from “introduction” to “conclusion”). Subsequent studies, currently conducted by the authors, strive to overcome this shortcoming by adding an observational, longitudinal element to interview accounts.

In summary, the present study aimed to develop an action framework for creative activity, one that strives to be more comprehensive than previous cognitive models of the creative process. This framework, both a theoretical and methodological tool, does not disregard earlier findings from the cognitive tradition but tries to integrate them into a more contextual perspective which reunites the psychological and behavioral aspects of creation with its material and social effects. As such, it strongly connects with contemporary “extended mind” theories (Clark and Chalmers, 1998) and a vision of cognition as distributed, external and situated. Applying this theoretical perspective results in “local” models that respect the particularities of each creative field, while enabling comparisons between them. These local models can also be very fruitful for our efforts to enhance creative expression in different domains, in educational settings and beyond.

## Conflict of Interest Statement

The authors declare that the research was conducted in the absence of any commercial or financial relationships that could be construed as a potential conflict of interest.
